# Supplementary material for: Are carbon emissions trading and green financial instruments synergistic? -Comprehensive quantitative research based on content analysis
Source: PLoS One. 2024 Mar 7;19(3):e0298601. doi: 10.1371/journal.pone.0298601 (PMC10919601; doi:10.1371/journal.pone.0298601)
Supplement: S1 Data — (ZIP) [file pone.0298601.s001.zip › Data/地级市Z_F工作报告.zip!/╔╜╢1⁄2/═■║ú╩╨/└┤╫╘╣1⁄2╓┌║┼í╛┬φ┐╦╩2╛▌═°í┐_2005.docx]

各位代表：

　　现在，我代表市人民政府，向大会作政府工作报告，请予审议，并请各位政协委员和其他列席会议的同志提出意见。

　　一、2004年政府工作的回顾

　　过去的一年，在省委、省政府和市委的正确领导下，市政府同全市人民一道，高举邓小平理论和“三个代表”重要思想伟大旗帜，以党的十六大和十六届三中、四中全会精神为指导，坚持以科学发展观总揽全局，深入贯彻中央加强宏观调控等一系列方针政策，认真落实市委确定的发展目标和工作思路，正确把握形势，努力破解难题，创造性地开展工作，圆满完成了市十四届人大二次会议确定的各项任务。

　　（一）经济发展速度明显加快。全市GDP突破千亿大关，达到1008.8亿元，比上年增长17.1%，其中一、二、三产业分别增长4.1%、21.1%和14.4%。实现财政总收入84.2亿元，增长40.7%，其中地方财政收入42.6亿元，增长42.9%；地方财政收入占GDP的比重、税收占地方财政收入的比重分别提高0.7和12个百分点。各项经济指标持续快速增长，全市用电量、货物周转量、港口吞吐量分别增长16.2%、66.7%和43.5%，主要指标提前完成“十五”计划，在向“翻三番”目标迈进中取得了历史性突破！

　　（二）对内对外开放迈出新的步伐。成功组织了欧洲经贸及旅游推介会、韩国招商月以及宁波、哈尔滨经贸洽谈会等50多次国内外招商活动，文化搭台、旅游推介、经贸唱戏等新的招商方式取得显著成效。按新口径计算，全年实际利用外资11.2亿美元，比上年增长38.8%；实际利用内资154.6亿元，增长60.2%。新引进总投资过千万美元的外资项目和过亿元的内资项目分别达到107个和80个，比上年分别增加48个和25个。外贸进出口总值达到56亿美元，增长38.8%，其中出口34.5亿美元，增长36.4%。

　　（三）结构调整成效显著。完成固定资产投资530.5亿元，比上年增长43.2%，其中工业投资253亿元，增长36.2%，一大批技术含量高、带动力强的产业项目建成投产，推进了“三大基地”建设，优化了产业结构，三次产业比重由上年的11.1：58.8：30.1调整为9.8：61.1：29.1。农业方面，调整种植业面积25.9万亩，粮食总产达到91.3万吨，与上年基本持平；肉、蛋、奶产量分别增长1.3%、4.5%和20.3%；远洋渔船由上年的101艘发展到139艘，水产品产量达到244.3万吨，继续保持全国领先地位；完成造林面积20.5万亩，林木覆盖率提高了2.2个百分点；林牧渔业占农业总产值的比重达到78%。工业方面，支柱产业和骨干企业快速膨胀，五大产业群完成增加值437亿元，增长27.7%；销售收入过10亿元的企业由上年的11家发展到26家，其中过30亿元的达到5家，工业“群山无峰”的局面开始改变。高新技术产业产值增长51.7%，占工业产值的比重提高了2.2个百分点。服务业方面，晚间经济开始活跃，消费市场更加繁荣，商业步行街、连锁超市等一批商贸设施投入使用，全社会消费品零售总额达到238.1亿元，增长15.2%。旅游业快速发展，实现总收入71.3亿元，增长35.6%。

　　（四）市场取向改革继续深化。企业上市取得重大突破，先后有威高、好当家等5家公司在国内外成功上市，募集资金13亿元。企业改革不断深化，县属以上和镇村办企业改制面分别达到93.5%和65%。开展了税收执法大检查，提高了税收征管质量。全面取消了农业特产税，降低农业税税率3个百分点，农民人均减负378元。推进了农村信用社改革，加大了金融安全区的创建力度。在全省率先推行了公务用车货币化改革。经省政府批准，设立了石岛管理区。成立了中小企业担保公司，组建了一批行业协会，民营经济快速发展，个体私营企业从业人员和实交税金分别增长56.1%和31.9%。

　　（五）发展环境进一步优化。成功举办了首届中国威海国际人居节、2004东北亚经济合作论坛及经贸洽谈会、亚洲科学园协会第八届年会等重要涉外活动，人居品牌更加突出，城市知名度和影响力有新的提高。加强了“诚信威海”和“平安威海”建设，威海信用网正式开通，安全生产和社会治安形势稳定好转，服务环境和法制环境有新的改善。围绕半岛城市群建设，编制了一体化威海战略规划和千公里海岸线控制规划；完成了市区九大城建重点工程，开展了城乡环境综合整治、威海湾海域清理等工作，狠抓了城市主干道、出入口等重点部位的绿化美化，生态市建设开局良好，城乡面貌发生新的变化。桑沟湾湿地成为全国第一个命名的“国家级城市湿地公园”，里口山风景名胜区成为全省第一个命名的“城市中的风景名胜区”。规范了土地市场秩序，撤销各类开发区47个。继去年国务院批准威海航空口岸对外开放后，经过多方面的精心准备，今年初又通过了通航前准备工作的国家验收，完全具备了对外开放的条件。与此同时，威乳高速公路、桃威铁路改建、威海港三期等重点工程亦进展顺利，立体化交通网络进一步完善。

　　（六）社会事业全面发展。深入实施“科教兴威”战略，全年共取得重要科技成果81项，其中达到国际领先水平10项；市级以上企业技术开发中心达到59个，比上年新增16个；三维数字城市地理信息系统应用项目通过国家验收，“数字威海”建设取得新的进展。进一步整合了市区职业技术教育和高中教育资源，新建改建中小学校舍19万平方米；高等教育有了新的发展，全日制本专科在校生达到2.3万人。完善了公共卫生体系，全市新型农村合作医疗覆盖面达到82.7%。实行了农村独生子女家庭奖励费财政统一发放制度，人口自然增长率连续6年保持负增长。争取了“心连心”艺术团来威演出，推出了《梦海》这一大型情景音乐剧，打造了文化旅游特色品牌，丰富了群众性文化活动。举办了7次省级以上体育赛事，全民健身活动更加普及。精神文明和民主法制建设成效显著。民兵预备役和“双拥”共建工作迈出新的步伐。广播电视、新闻出版、民族、宗教、外事、侨务、人防、史志、档案、气象、地震、妇女儿童、老龄、残疾人等事业都取得了新的成绩。

　　（七）人民生活水平有新的提高。就业再就业工作进一步加强，全年新增城镇就业人口4.96万人，转移农村劳动力1.6万人，城镇登记失业率控制在1.7%以内。社会保障体系更加完善，城镇职工养老、医疗、失业、生育、工伤保险覆盖面均保持在98%以上。强化了社会救助体系建设，出台了城市最低生活保障对象医疗教育住房救助办法，提高了最低工资标准，弱势群体得到有效救助。儿童福利院一期、经济适用住房三期工程已投入使用，老年公寓二期已完成主体框架工程，解决了市区居民小区和城中村路灯照明问题，完成了天然气置换，新增通气用户3660户，新增供热用户6000户，社区基础设施、服务网络不断完善。建立了失地农民基本生活保障制度、建筑行业农民工工资保障金制度，依法查处了拖欠克扣职工工资等违法侵权行为，群众关心的一些热点难点问题得到有效解决。城镇居民人均可支配收入达到11112元，增长9%；农民人均纯收入达到5376元，增长13.1%；年末城乡居民储蓄存款余额394.9亿元，比年初增长10%。消费结构加速升级，汽车、住房、旅游、教育等成为新的消费热点，人民群众的生活质量明显提高，思想观念和精神风貌也发生积极变化。

　　（八）政府自身建设不断加强。按照市委的统一部署，各级政府开展了新一轮思想解放活动，主动找差距，自觉加压力，着力解决了影响发展的思想观念、领导方式和工作作风等方面的问题。围绕建设法治政府、阳光政府和责任政府，深入贯彻实施《行政许可法》，对原有行政许可事项、许可主体及许可收费项目进行了全面清理；定期向人大和政协通报政府工作情况，认真办理人大代表建议和政协提案，邀请部分人大代表、政协委员及社会各界人士列席市政府全体会议；强化了决策目标、执行责任、考核监督“三个体系”建设，提高了政府工作效能；完善了市长公开电话和行风热线，开通了市长电子信箱，强化了纠风工作，查处了违法违纪案件，政风行风有新的转变；各级政府主要领导同志都公开向社会做出了廉政承诺。

　　各位代表！过去的一年，是不平凡的一年。面对新的形势和日趋激烈的国内外竞争，全市上下高扬加快发展的主旋律，发挥比较优势，实施重点突破，经济发展跨上新台阶，城乡面貌发生新变化，人民生活有了新提高，各项事业迈出新步伐。荣成市获得了“中国魅力城市”称号，文登市被授予“中国工艺家纺名城”称号，乳山市跨入“全国百强县”行列，三市一区在全省第六届精神文明建设先进县市区评比中继续保持“一片红”，我市的城市综合竞争力也由全国第42位跃升到第36位。取得这样好的成绩，我们倍感鼓舞和自豪，同时也深知这大好局面来之不易，每一个成果的取得，每一项事业的成功，无不凝聚着同志们、朋友们的心血和汗水。在此，我代表市人民政府，向全市所有建设者和所有纳税人，向各位人大代表和政协委员，向各民主党派、工商联、无党派人士和社会团体，向驻威部队指战员、武警官兵和政法干警，向各位老领导、老同志，向所有关心支持威海建设的港澳台同胞、海外侨胞和国际友人，致以崇高的敬意和衷心的感谢！

　　各位代表！回顾一年来的工作，我们深切体会到，要不断开创改革开放和现代化建设的新局面，必须根据形势的要求和发展的需要，不断解放思想、更新观念，在与时俱进、抢抓机遇中创造性开展工作；必须坚持用科学发展观统揽全局，适时把经济发展的着力点转到调整结构、深化改革、转变增长方式上来，集中力量解决自身发展中存在的突出矛盾和问题，推进经济社会统筹协调发展；必须坚持以人为本，着力解决群众关心的热点、难点问题，让人民群众在改革和发展中得到更多实惠；必须在各级党委的领导下，坚持科学行政、民主行政、依法行政，不断加强政府自身建设，努力提高行政能力和水平；必须保护好、引导好、发挥好方方面面的积极性，营造你追我赶、干事创业、加快发展的浓厚氛围。这些都是我们开拓创新的宝贵经验，是我们乘势而上的坚实基础，是我们加快发展的精神财富，一定要在今后的工作中发扬光大。

　　各位代表！在总结成绩的同时，我们也清醒地看到，我市经济社会发展中还存在一些突出问题。主要是：对科学发展观的认识需要进一步深化，工作措施有待进一步加大；结构性矛盾依然突出，“三农”工作特别是农民持续增收还缺乏有力的保障，工业“群山无峰”的局面还没有根本性改变，服务业发展还相对滞后；各类急需人才仍十分短缺；财税的“三个比重”，特别是两税收入占二、三产业增加值的比重还较低；土地的集约化利用水平不高，园区配套水平还需进一步提高；少数群众的生产生活还比较困难，经济与社会发展还有不相协调的地方；少数部门的工作作风、行政效能有待进一步改善，腐败现象和不正之风还时有发生，等等。对此，我们一定保持清醒头脑，正视差距和不足，采取切实措施认真加以解决。

　　二、2005年经济社会发展的总体要求和工作重点

　　今年是全面完成“十五”计划、衔接“十一五”发展的关键一年。做好今年的工作，对保持经济社会良好发展势头、实现本世纪第一个翻番，具有十分重要的意义。按照省委、省政府的部署和市十二届三次党代会的精神，今年政府工作的总体要求是，以邓小平理论和“三个代表”重要思想为指导，全面贯彻十六大和十六届三中、四中全会精神，牢牢抓住发展这个第一要务，用科学发展观统领经济社会发展全局，积极贯彻落实中央宏观调控政策，坚定不移地实施市委确定的发展目标和工作思路，更好地利用重要战略机遇期，加快“三大基地”建设，在结构调整、改革开放、生态建设、构建和谐社会和政府自身建设等方面取得更大成效，不断提高人民群众的物质文化生活水平，努力实现经济社会更快更好发展。主要预期目标是：全市GDP增长15%以上；地方财政收入增长20%，力争25%；固定资产投资增长20%，力争25%；外贸出口总值增长20%，力争25%；实际利用外资增长20%，力争25%；实际利用内资增长25%，力争30%；社会消费品零售总额增长13%；城镇居民人均可支配收入增长8%；农民人均纯收入增长6%；城镇登记失业率控制在2%以内；人口自然增长率控制在2‰以内；各市区在全国、全省的位次都要努力前移。

　　各位代表，新的一年，我们面临全面发展的良好机遇和有利条件，世界产业加速向中国转移势不可挡，国内经济自主增长活力明显增强，胶东半岛制造业基地和山东半岛城市群战略的全面实施等，为威海的腾飞提供了广阔空间。从自身看，全市上下加快发展的积极性更加高涨，发展环境更加优化，基础设施更加完善，加之前两年加大投入形成的产能将在今明两年逐渐得以释放，使得我们完全有条件发展得更快更好一些。同时也要看到，随着国际贸易摩擦的增多，区域竞争的加剧，要素供给矛盾的加大，我们加快发展的外部环境、支撑条件、增长动力和方式都将发生新的变化。我们必须坚持辩证思维，化不利为有利，变挑战为机遇，发扬市十二届三次党代会提出的“自强不息，创新创业，海纳百川，追求卓越”的威海精神，进一步解放思想，坚定信心，乘势而上，圆满完成全年的预期目标，确保全市经济社会发展始终充满生机和活力，始终保持更快更好的发展势头。工作中，要切实抓好以下十个重点：

　　（一）着力提高开放的层次和水平。从全球化视野出发，紧紧抓住国际产业转移、国内南资北上的有利机遇，在更大范围、更宽领域、更高层次上构筑全方位开放格局。一是提高招商引资的规模和质量。继续坚持主攻韩日、扩大台港、开拓欧美的方针，精心组织好韩日招商月、文化经贸活动周以及欧美经贸促进、港台招商等重要涉外推介活动；坚持以长三角、珠三角及东北等地区为重点，组织好杭州、沈阳、温州等经贸合作周活动，加快建立重点带动、多元发展的国内外招商格局。要认真创新招商方式，提高招商针对性，注重招商实效，降低招商成本。要按照文化搭台、旅游推介、经贸唱戏的思路，整合和利用好招商资源，加快建立以企业为主体、以中介组织和专业队伍为依托的招商新机制。要以大企业、大项目为重点，引进一批高新技术和大型基础性、装备性项目，鼓励外资投向农业、服务业和基础设施、城市公用设施领域，引导外资企业把技术含量高、增值大的加工环节和研发、中试机构转移到我市，不断提升利用外资的层次和水平。二是继续搞好开发区和工业园区建设。开发区是我市开放的龙头，一定要加大培植力度，努力使其成为现代制造业聚集的新型工业区、资源合理利用的示范区和统筹协调发展的新型社区。高技术产业开发区要突出高新技术项目的载体功能，搞好与高校的联合共建，大力推进科技新城建设，不断提升园区的科技创新能力。经济技术开发区要突出临港优势，搞好临港工业区和出口加工区的开发建设，不断提升承接能力。要突出抓好草庙子工业园的规划建设，引导工业企业向园区集中，做大制造业规模，以此为依托全面加快市区与文登城区的对接。其它各类园区也要进一步突出特色，优化布局，形成群体优势和集聚效应，避免要素分散、资源浪费和重复建设，切实提高投资强度和产出水平。三是不断加大市场开拓力度。牢固树立国内国外统一大市场的理念，引导企业积极参加国内外知名贸易展销活动，进一步扩大市场份额。深入实施“科技兴贸”、“以质取胜”战略，鼓励企业获得国际通行的产品质量、环境质量和安全标准体系认证，增强市场竞争力。引导加工贸易升级转型，进一步优化出口产品结构。坚持进出口并举，扩大重要物资、原材料和先进技术、关键设备进口。全年机电产品和高新技术产品出口比重提高到40%和24%以上。四是提高应对国际贸易摩擦的能力。要抓紧做好入世过渡期结束后的各项对策准备，建立和完善外经贸预警机制，协助企业做好知识产权调查、反倾销、反补贴和技术贸易壁垒的应对工作。五是积极实施“走出去”战略。支持和组织有条件的企业，联合、联片开发国外、市外战略资源，实施低成本扩张。继续搞好对外工程承包和劳务合作，鼓励企业积极承揽境内外大型工程项目。

　　（二）加快推进经济结构的战略性调整。坚持工业强市、三产兴市，不断优化产业结构，构筑更具竞争力的现代产业体系，全力提高经济运行质量和效益。

　　围绕制造业基地建设，加速工业结构优化升级，尽快形成一批支柱产业、一批大型企业和一批知名品牌。一是大力发展集群经济。要以技术改造为突破口，以提高产业配套能力和水平为重点，加快高新技术和先进适用技术向传统产业扩散和渗透，进一步拉长产业链条，培植壮大五大产业群。要坚持以信息化带动工业化，大力推广“项目、基地、人才”一体化模式，积极发展电子信息、生物技术、新材料和海洋技术等新兴产业，大力培植有前途的高科技项目，加快形成具有地方特色的高新技术产业群。年内五大产业群增加值增长20%以上，高新技术产业产值比重提高2个百分点以上。二是做大做强骨干企业。制订重点企业做大做强攻坚计划，加大对市级重点企业的扶持力度，形成你追我赶的争峰局面。按照抓两头，带中间的方法，搞好资产重组，引导稀缺资源向优势企业集中，尽快做大一批企业集团，工业“群山无峰”的局面要有新的转变。鼓励企业加大技术改造投入，确保三角和成山子午胎、蓝星系列玻璃、威高血液净化产品、光威精密压铸模具等100个投资过亿元的工业项目完成年度投资计划。同时，要创造条件，鼓励和支持有发展前景的中小型高新技术企业加快成长壮大，形成新的经济亮点。三是积极实施品牌战略。鼓励和引导企业在扩张规模的同时，以一流的管理、一流的产品和一流的服务，打造一流的品牌。支持有条件的企业开展品牌经营，放大品牌效应，走出一条以人才兴科技、以科技上质量、以质量拓市场、以市场树品牌的科学发展之路。今年争取在申报中国驰名商标和中国名牌产品上有新的突破。

　　围绕旅游度假基地建设，加快培植新的服务业亮点，推动服务业扩规模、上档次。一是加快发展旅游业。围绕省里提出的“山水圣人”和“黄金海岸”两大旅游体系建设，搞好旅游资源整合与深度开发，加快千公里“幸福海岸线”建设步伐。集中力量抓好刘公岛海权文化主题公园、福如东海、温泉疗养、游艇俱乐部等重点旅游项目建设，打造一批特色旅游品牌。积极开发参与性、度假性旅游新品种，推动观光旅游向休闲度假旅游延伸。全年旅游人数和总收入增长15%以上。二是大力发展会展经济。坚持突出特色和市场运作，加快国际商品交易中心建设，高水平办好第二届国际人居节，打造会展经济的品牌，推进会展业与旅游、商贸等产业有机结合和相互促进。三是着力提升服务业整体水平。在改造提升传统服务业的同时，坚持生产和生活并举，加快服务业信息化、自动化、智能化步伐，积极发展金融信贷、信息咨询、电子商务、社区服务等现代服务业。港口是我市的一大优势，必须积极实施“以港兴市”战略，进一步搞好港区规划，整合港口资源，大力发展临港工业和港口物流业。要制定优惠政策，扶持服务企业做大做强，尽快培植一批规模大、知名度高的名牌企业。

　　（三）千方百计做好“三农”工作。在中央1号文件精神的指引下，以建设海产品为主的农副产品出口加工基地为重点，以农民增收为核心，以结构调整为主线，按照“以城带乡、以工促农”和“多予、少取、放活”的要求，统筹抓好各项农村工作，切实提高农业综合生产能力。一是加大农业结构调整力度。在加强基本农田保护、确保粮食生产安全的基础上，大力发展以花生、果品、蔬菜、药材为支柱的特色种植业，巩固提高以奶业为龙头的特色畜牧业，努力构建优质高效农业产业带。年内调增经济作物20万亩，奶类总产量增长15%以上。海洋是我市经济发展的独特优势，要根据资源现状和产业趋势，加快发展以远洋捕捞、名优养殖、海产品加工、海洋旅游等为重点的新型海洋产业，提高海洋综合开发水平，实现渔业经济向海洋经济的转变。二是加快农业产业化、标准化、国际化步伐。大力扶持龙头企业和农村中介组织，加快标准化基地建设，健全农产品质量安全监测和动植物疫病防治体系，积极发展外向型农业，努力探索农业规模化、集约化经营的新模式和新途径。年内标准化生产基地发展到80万亩以上，农副产品出口增长25%以上。三是全面落实各项扶农政策。继续深化农村税费改革，年内全部取消农业税。认真落实农村干部工资财政补贴办法，清理化解镇村两级债务，切实减轻农民负担。建立健全良性投入机制，加大财政支农力度，扩大信贷支农规模，改造中低产田，提高水利、农机等农业基础设施投资比重。四是统筹城乡协调发展。推动各类规划向镇村延伸，加快小城镇建设，鼓励有条件的村庄适度集中进行旧村改造，促进土地集约利用。以搞好“路水电医学”建设为基础，综合整治农村环境。建立完善农村劳动力就业服务体系，引导农民向城镇、向二三产业有序转移，全年实现转移就业2万人。

　　（四）深入推进各项改革。以改革年为契机，围绕解决制约发展的体制性、机制性障碍，全面推进各项改革，力争在一些重点领域和关键环节取得大的进展。一是继续深化企业改革。坚持把企业改制同资产重组、结构调整、招商引资结合起来，以建立现代产权制度为重点，突出抓好困难企业改制和解困工作，年内县属以上企业基本完成改制任务，镇村办企业改制面达到80%以上。加强对改制企业的指导，进一步完善法人治理结构，实现规范运作。二是搞好各项配套改革。健全公有资产监督管理体制，完善授权经营、外派监事制度和企业经营业绩考核制度，确保公有资产保值增值。稳妥推进投资体制改革，凡是符合国家法律和产业政策的非政府投资项目，不再实行审批制。继续加大创建金融安全区的工作力度，不断深化农村信用社改革，完善金融服务体系。加强建设用地的审批管理，进一步规范经营性用地招标、拍卖、挂牌出让制度，提高土地集约利用水平。加快事业单位改革步伐，继续搞好城市供水、供热、园林、公交等市政公用行业的改革，逐步建立政事职责分开、法人地位明确、单位自主经营、政府依法管理的新型管理体制和运行机制。培育和规范市场中介组织，促进各类要素合理流动、充分竞争。三是加快发展民营经济。认真落实各项扶持政策，进一步降低门槛，着力创造平等竞争、一视同仁的创业环境。按照政府引导、市场运作的原则，加快民营经济融资担保、信用评价、人才培训、技术信息、法律服务和行业协会六个服务体系建设，培育一批跨地区、跨行业、跨所有制的大型民营企业集团。

　　（五）保持投资和消费稳定增长。坚定不移地贯彻落实中央宏观调控政策，按照区别对待、有保有压的原则，优化投资结构，确保有效投入快速增长、投资效益不断提高。认真研究上级的投资导向，筛选一批水平高、带动力强的大项目，争取进入国家、省计划盘子。高度重视和支持金融工作，完善担保体系，推动银企互动，引导金融机构调整信贷投向，增加对重点项目的信贷投放。狠抓企业上市工作，确保4家、力争6家公司在境内外发行上市和再融资。搞好三角、海马等12家企业的上市辅导，形成梯次上市格局。积极启动民间资本，鼓励本地企业扩大投资。要突出抓好市级重点项目建设：产业领域，重点抓好威海船厂搬迁扩建、渔港码头搬迁等项目；基础设施领域，重点抓好威乳高速公路、刘公岛空中客车等项目，并力争莱荣高速提前开工，烟威荣铁路尽快立项；便民利民领域，重点抓好市区、开发区中小学校建设等项目。同时，抓好各市区筛选的重点项目，争取早开工、早投产、早见效。

　　市场经济条件下，消费是拉动经济增长的最重要力量。要合理调整投资与消费的关系，积极扩大消费需求，不断增强消费对经济的拉动作用。健全与经济发展水平相适应的收入增长机制，努力增加城乡居民收入，提高居民购买力。启动中心商业区改造工程，进一步优化各类商业网点布局，构建方便快捷的社区商贸服务体系。引导、扶持优势企业向农村延伸网点，建立健全农村商贸网络，进一步活跃农村市场。继续发展配送中心、连锁经营、专卖店、专业店等现代流通业态，进一步繁荣晚间市场，扩大消费信贷，推动消费结构升级。同时，要整顿规范市场秩序，深入开展食品、药品、烟草、交通、房地产等专项治理，严厉打击见利忘义、假冒伪劣、掺杂使假、坑害群众等不法行为，确保人民群众消费安全。

　　（六）大力推进生态市建设。坚持生态建设与环境整治一齐抓，在更高层次上推进城乡建设，进一步打响人居品牌。一是搞好城乡规划建设。充分发挥规划的先导作用和约束力，认真落实“一带两线”的总体规划，切实加强各类规划的编制工作，不断扩大城市详规覆盖率。以建设世界精品城市为目标，不断强化精品意识，提高项目的功能质量和艺术含量。中心城市要重点抓好环翠楼公园改造、昆明路隧道延伸、污水厂和污水管网敷设等11项城建重点工程，努力提升城市品位。以方便群众生活为目标，搞好社区建设，不断提高城市服务功能，让市民成为城市建设的最大受益者。以打造一体化威海为目标，加速城市板块之间的衔接，加大对石岛管理区的支持力度，使其尽快成为经济强区、精品城区、旅游名区和文明社区，成为威海的一大重要板块。其他次中心城市和中心镇都要按照增强功能、优化环境、突出特色的思路，规划建设好各自的精品工程。二是大力推进生态市建设。搞好《生态市建设总体规划》的编制和实施，广泛开展“碧海、蓝天、青山”行动，争创全省第一个国家级生态示范市。全面落实环境保护市长目标责任制，严格执行环境影响评价和“三同时”制度，搞好以背街小巷、城中村为重点的城乡环境综合整治，加快各类治污设施建设，从源头上控制新的环境污染。加强资源开发监督管理，适度控制矿山开发。增强海洋国土意识，加大对海岸带的管制力度，搞好海湾综合治理，保护海洋环境。围绕争创全国首批“生态园林城市”，继续实施“五林”工程，优化树种结构，搞好烟威路、桃威铁路绿化和环海路、机场路两侧可视范围荒山的绿化美化，年内完成造林20万亩，林木覆盖率再提高2个百分点。三是加快节约型社会建设。大力发展循环经济、生态经济，抓好海水淡化、废水回用、废渣综合利用等项目的实施，淘汰落后工艺，推行清洁生产，把加快发展建立在资源环境承载能力的基础上。大力提倡绿色消费，努力在全社会形成节约资源的理念和生活方式。

　　（七）积极做好财税工作。大力培植财源，抓好增收节支，继续保持收支平衡，不断提高地方财政收入占GDP的比重、税收占地方财政收入的比重、两税收入占二三产业增加值的比重，使经济发展的成果真正反映到财政收入上来。要把“三个比重”纳入对各级的考核体系，与GDP挂钩，与对下的转移支付挂钩。要坚持依法征税，完善征管体制，强化税基控管。大力推进综合治税，加强税收源头管理，严厉打击各种偷逃骗税行为，切实做到应收尽收。认真清理规范税收优惠政策，加大对税收征管的监督检查力度，坚决制止有税不收或收过头税。要进一步强化非税收入管理，不断优化收入结构。要落实好省里扶持经济强县的政策措施，加快县域经济发展，确保县镇财政收入大幅度增长。要按照转变政府职能的要求，继续优化支出结构，建立与经济社会发展相适应的公共财政体制。进一步深化财政预算编制改革，积极完善财政监督机制，切实维护预算的严肃性。建立健全政府投资评审机制，实施预算内投资项目代建制和委托投资咨询评估管理办法，健全政府投资后评估和投资决策责任追究制度，不断提高财政资金的使用效益。

　　（八）统筹发展各项社会事业。一是全面推进科技创新。以“863”计划成果产业化基地和威海国际生物、国际微电子研发中心为依托，加大科研投入，组织研发攻关，加快碳纤维原丝、电动汽车等重大科技项目的产业化进程。加强中介服务体系建设，大力开展产学研联合，引导企业建立技术创新体系和成果转换机制。认真做好信息化试点市工作，继续推进“数字威海”建设。二是加快教育事业发展。继续巩固义务教育，加快普及普通高中教育。加大教育投入，搞好农村中小学校危房改造，加快市区中小学校舍建设，进一步改善办学条件。支持驻威高校和职业技术学院发展，积极引进和联办高等院校。三是深入实施人才强市战略。坚持把促进发展作为人才工作的根本出发点，充分认识人才对提高我市发展水平和层次的极端重要性，加快高层次人才和各类急需专门人才的培养和引进，加强高技能人才队伍建设。完善人才政策，创新工作机制，努力营造优秀人才脱颖而出、人尽其才的有效机制和良好环境。四是大力发展卫生事业。全面完善公共卫生两个体系建设，抓好重点传染病预防控制，高水平全面完成市级传染病院建设，进一步提高公共卫生服务水平。继续搞好社区卫生服务体系和农村卫生院建设，巩固完善新型农村合作医疗制度，争取各市区全部列入省级合作医疗试点范围。五是积极发展文化体育等其他各项社会事业。大力发展群众性文化，广泛开展全民健身活动，进一步丰富群众生活。运用市场化手段，进一步完善《梦海》剧目，创出我市文化旅游的新品牌。组织一批国际、国内专项体育比赛，把文化、体育产业的发展与旅游业有机结合起来。加快广播电视事业发展。深入开展科技、文化、卫生“三下乡”活动。高度重视人口和计划生育工作，积极应对第四次人口出生高峰，加快人口现代化。进一步做好妇女儿童、老龄和档案、史志、气象、测绘、防震减灾等各项工作。做好民族、宗教、对台和侨务工作。切实搞好国防教育、国防动员、人民防空和民兵预备役建设，做好转业军人和退役士兵转退安置，更加扎实地开展“双拥”共建活动。

　　（九）解决好关系群众切身利益的问题。一是积极扩大就业。把就业和再就业工作放在更加突出的位置，全面落实各项就业扶持政策，加强就业服务体系建设，加大对就业目标责任制的考核力度，不断扩大就业容量，年内新增城镇就业4.8万人。规范企业用工行为，落实最低工资标准和调整制度，切实维护职工和进城务工人员的合法权益。二是完善社会保障体系。继续扩大社会保险覆盖面，年内养老、医疗、工伤保险人数各增加2万人以上。加大社保基金征缴和监管力度，搞好企业退休人员社会化管理服务，确保养老金按时足额发放。全面建立失地农民基本生活保障制度，积极完善农民社会养老保险制度。三是加强社会救助体系建设。更加重视和关心社会弱势群体，切实帮助他们解决实际困难。进一步提高城乡居民最低生活保障标准，落实农村五保供养政策，搞好经济适用住房建设，加强医疗、教育、住房等专项救助。积极发展慈善和社会福利事业，制定完善城乡救灾应急预案，做好受灾群众生产生活救济工作。大力发展残疾人事业，加快推进残疾人的健康、就业、奔小康工作。同时，进一步增加公共支出，逐步解决地区之间和部分社会成员之间收入差距过大的问题，努力维护和实现社会公平。

　　（十）积极构建社会主义和谐社会。一是加强社会主义精神文明建设。扎实推进文明城市、文明村镇、文明行业创建活动，争创全国文明城市。大力弘扬民族精神，倡树威海精神，加强思想道德建设，不断提高公民文明素质。宣传普及现代科学知识，倡导健康文明的生活方式，努力提高全社会文明程度。以威海信用网为依托，以建立公务员承诺制度、企业信用评价和监督管理制度、个人信用制度为重点，加快建立社会信用体系，努力建设“诚信威海”。二是高度重视民主法制建设。各级政府要自觉接受人大及其常委会的监督，主动加强与政协的联系，广泛听取各民主党派、工商联、无党派人士及各人民团体的意见建议，认真办好人大代表建议和政协提案。进一步完善基层群众性自治组织和企事业单位的民主管理制度，积极推进政务公开、厂务公开和村务公开，保障人民群众依法行使民主选举、民主决策、民主管理、民主监督的权利。深入开展普法工作，健全法律援助体系，提高全社会依法治理水平。三是全力维护社会稳定。坚持重心下移，切实加强社会治安综合治理，严厉打击各类刑事犯罪，深入推进“平安威海”建设。重视和加强人民来信来访工作，完善化解人民内部矛盾的工作机制，及时排查调处社会矛盾纠纷，把问题解决在基层和萌芽状态。充分发挥仲裁、调解的作用，妥善处理与人民群众切身利益相关的各种问题。牢固树立“安全第一”和“责任重于泰山”的观念，认真落实安全生产各项规章制度和保障措施，坚决防止重大安全事故的发生。

　　各位代表！除了做好以上工作外，今年还有一项重要任务，就是抓紧做好“十一五”规划的编制工作。各级政府务必高度重视，把以人为本、全面协调可持续的发展理念贯穿规划始终，把增进人民福利、促进社会和谐放在重要位置，把促进城乡、区域、内外源型经济、人与自然、经济与社会等协调发展作为主要任务，认真调查研究，深入分析论证，广泛征求意见，充分集中民智，力求使新一轮规划具有较高的预见性、科学性和指导性。

　　三、进一步加强政府自身建设

　　今年的任务繁重而艰巨。各级政府必须适应新形势新任务的要求，深刻理解和正确把握党的十六届四中全会精髓，坚持为民、务实、清廉，不断加强自身建设，努力建设党和人民满意的政府。

　　着眼于保持党的先进性，切实加强政府公务员的思想政治建设。各级政府要按照市委统一部署，深入开展保持共产党员先进性教育活动。要组织广大公务人员认真学习邓小平理论和“三个代表”重要思想，深刻把握党的十六大和十六届三中、四中全会精神实质，牢固树立科学的世界观、人生观、价值观，增强贯彻执行党的基本理论、基本路线和基本纲领的自觉性，增强政治敏锐性和鉴别力。要注重理论和实践相结合，不断掌握新知识，积累新经验，增长新本领，努力提高为人民服务的能力和水平。

　　坚持依法行政，着力强化“三个体系”建设。要进一步完善决策目标、执行责任、考核监督“三个体系”，从制度和机制上调动和保护公务员干事创业的积极性、主动性和创造性。要认真学习贯彻《全面推进依法行政实施纲要》，增强按市场经济规律管理经济的意识和能力，增强按国际通行规则办事的意识和能力，增强主要运用经济、法律手段调节经济运行的意识和能力，加快政府职能转变。要把科学决策、依法决策、民主决策作为政府决策的基本准则，健全决策规则，规范决策程序，强化决策责任，进一步完善群众参与、专家咨询和政府决策相结合的决策机制，尤其是对专业性、技术性较强的决策事项，要认真组织专家论证和决策评估。继续邀请人大代表、政协委员、企业代表和基层群众参加政府重要会议。要深入贯彻《行政许可法》，健全行政许可各项配套制度。对依法保留的行政许可项目原则上全部纳入行政审批中心集中办理，进一步提高办事效率。要大力推进行政执法责任制和责任追究制度，对超越权限或违反法定程序造成重大损失的决策行为，必须严肃追究决策者的责任；对因违法行政给群众带来的损害，必须依法予以赔偿；对因政策调整给群众带来的损失，必须依法予以补偿。要客观、公正地考核政府工作人员在不同地区、不同基础条件下取得的实绩，横向比较看位次，纵向比较看变化，增强考核工作的科学性。

　　坚持执政为民，密切与人民群众的血肉联系。各级领导干部要坚持立党为公、执政为民，牢固树立正确的政绩观，常修为政之德，常思贪欲之害，常怀律己之心，切实把一切工作的成果都体现在让人民群众享受到实实在在的利益上。要大兴调查研究之风，深入基层，特别要到条件差、困难大、群众意见多的地方去，体察民情，解决问题，今年要继续解决好征用土地、城镇房屋拆迁、企业违法排污、企业重组改制和破产中损害群众利益，以及拖欠农民工工资等突出问题，千方百计把广大人民群众的根本利益实现好、维护好、发展好。

　　坚持务实高效，进一步转变工作作风。各级政府都要把抓落实作为首要任务，坚持重实际、说实话、办实事、求实效，下决心精简会议和文件，坚决防止和克服官僚主义、形式主义，反对弄虚作假、虚报浮夸，决不搞脱离实际、劳民伤财的“政绩工程”、“形象工程”。要按照定目标、定领导、定时间、定责任的要求，健全快捷高效的抓落实工作机制，对市委、市政府确定的各项发展战略、措施和目标，要层层分解，逐级落实，严明奖惩，确保各项工作一抓到底，抓出成效，干出水平。要严格执行《统计法》、《审计法》，推进统计改革和建设，加强经济审计监督。

　　坚持从严治政，切实加强廉政建设。要严格按照全国落实党风廉政建设责任制工作会议提出的“五不许”要求，加大从源头上预防和惩治腐败的工作力度。认真办好12345市长公开电话和行风热线，搞好行风评议，加大行政效能监督力度，坚决纠正部门和行业不正之风。要抓好政府采购、“收支两条线”管理，健全建设工程项目公开招标和产权交易进入市场制度。每一位政府工作人员，都要以清廉自律、艰苦奋斗的作风，体现党的宗旨，塑造政府良好形象，对违法乱纪、贪污受贿、徇私枉法和严重侵害国家、集体和群众利益的案件，要坚决查处，决不姑息。

　　各位代表！肩负着“翻三番”的时代重任，我们又踏上了新的征程。让我们紧密团结在以胡锦涛同志为总书记的党中央周围，高举邓小平理论和“三个代表”重要思想伟大旗帜，在中共威海市委的领导下，坚定信心，振奋精神，抓住机遇，扎实工作，继续保持经济社会持续快速健康发展的大好势头，为加速建设全面小康和现代化新威海做出新的更大的贡献！
